# A Novel Method for Detecting Fe^2+^ at a Micromolar Concentration Based on Multiple Self-Mixing Interference Using a Fiber Laser

**DOI:** 10.3390/s23052838

**Published:** 2023-03-05

**Authors:** Wu Sun, Zhuo Yang, Guo Feng, Zhou Chen, Qiaoyun Chang, Lan Hai, Zeqing Guo

**Affiliations:** Key Laboratory of Functional Materials and Devices for Informatics of Anhui Educational Institutions, Fuyang Normal University, Fuyang 236037, China

**Keywords:** laser self-mixing interference, absorption decay, micromolar concentration, external cavity

## Abstract

The concentration of an electrolyte is an optical characteristic of drinking water. We propose a method based on the multiple self-mixing interference with absorption for detecting the Fe^2+^ indicator as the electrolyte sample at a micromolar concentration. The theoretical expressions were derived based on the lasing amplitude condition in the presence of the reflected lights considering the concentration of the Fe^2+^ indicator via the absorption decay according to Beer’s law. The experimental setup was built to observe MSMI waveform using a green laser whose wavelength was located in the extent of the Fe^2+^ indicator’s absorption spectrum. The waveforms of the multiple self-mixing interference were simulated and observed at different concentrations. The simulated and experimental waveforms both contained the main and parasitic fringes whose amplitudes varied at different concentrations with different degrees, as the reflected lights participated in the lasing gain after absorption decay by the Fe^2+^ indicator. The experimental results and the simulated results showed a nonlinear logarithmic distribution of the amplitude ratio, the defined parameter estimating the waveform variations, versus the concentration of the Fe^2+^ indicator via numerical fitting.

## 1. Introduction

Laser self-mixing interference (SMI), a remarkably universal phenomenon [1,2,3], has been popular in recent years [4,5,6,7]; it occurs when the emitting light is re-injected into the laser cavity after reflecting or back-scattering caused by the external reflector [8,9,10,11,12]. As a result, the re-injected beam modulates the laser characteristics, such as the laser intensity. Therefore, modulated laser intensity appears as a periodical cosine function consisting of several fringes in the time domain at a weak feedback regime [2,3]. Laser self-mixing interference (also as known as the laser feedback effect) has been widely applied to measure physical parameters, such as velocity [13,14,15,16]., displacement [17,18,19], vibration [20,21,22], distance [23,24,25,26], flow [27,28,29,30], angle [31,32,33,34], and refractive index [35,36].

The multiple reflections in an external cavity can often be ignored if there is a rough reflector, such as white paper, that makes it difficult to generate multiple reflections in such an external cavity. This is because only part of the light is able to re-enter the laser cavity with a finite facet while the diffusive effect causes that to be much larger than the laser facet as the rays of the diffusive light propagate in different directions. Provided that the surface of the external reflector is smooth (e.g., a plane mirror), the reflector is able to generate multiple reflections in the external cavity since specular reflection makes the rays of the reflected light propagate in the same direction. With multiple reflections of the light in the external cavity within the laser output facet and the smooth reflector, the reflected lights will be able to re-enter the laser cavity, with every reflection causing multiple laser self-mixing interference (MSMI) [37,38,39,40,41], whose fringe numbers will be folded corresponding to the reflection orders. Every reflected beam re-injected in the laser cavity will result in the superposition of the fringes in the time domain.

In this work, it was observed that the absorption decay by absorbent solutions in the external cavity could noticeably affect the MSMI waveform in the time domain via the reflected lights. The intensities of the reflected light would decrease to various degrees, and the decrement degree could be related to both the reflection orders and the material concentration for a specific electrolyte, such as Fe^2+^. The MSMI waveform contained the main fringes and parasitic fringes that captured the absorption decay, as every reflected light’s intensity corresponded to the amplitudes of the fringes. The main fringe and parasitic fringe usually experienced different decrements, and the difference was related to the material concentration.

We propose a novel method for detecting Fe^2+^ at a micromolar concentration based on multiple self-mixing interference using a fiber laser. The laser intensity equations were derived considering the multiple reflected lights absorbed by the absorption material in the external cavity based on lasing conditions, and the waveforms were simulated according to the derived equation at different concentrations of the Fe^2+^ indicator. The experimental setup was constructed to observe the MSMI waveform using a green laser whose wavelength was located in the extent of the Fe^2+^ indicator’s absorption spectrum. The MSMI waveforms around the micromolar concentrations of the Fe^2+^ indicator were obtained in the time domain experimentally. Both the simulated and experimental waveforms contained the main and parasitic fringes, and a parameter A_2_/A_1_ indicating the amplitude ratio of the parasitic fringes versus the main fringes was defined to determine the distribution between the concentration and the MSMI waveforms using the numerical fitting, specifically, by estimating the R^2^ of the fitting.

## 2. Materials and Methods

**Figure 1:**
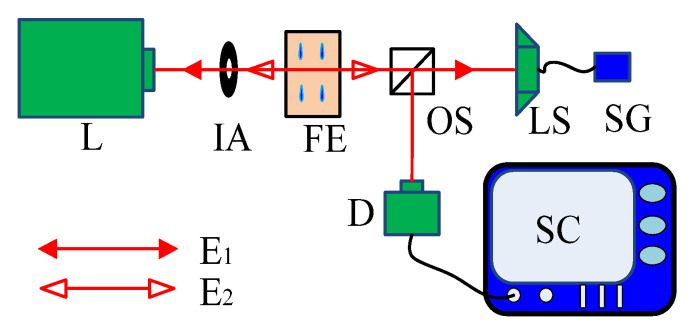
Scheme of the experimental setup (L: laser, IA: iris aperture, FE: a cuvette of Fe^2+^ indicator, OS: optical splitter, D: detector, SC: oscilloscope, LS: loudspeaker with a reflector, and SG: signal generator).

As shown in Figure 1, the experimental system consisted of a laser (L: 532 nm), an iris aperture (IA), a cuvette with the Fe^2+^ indicator sample (FE), an optical splitter (OS), a photoelectric detector (D), a loudspeaker (LS), and a signal generator (SG). A cuvette, a type of highly transparent container that guaranteed the laser can pass through without decay, was employed to change the absorption decay in the external cavity by diluting the Fe^2+^ indicator. The loudspeaker with a high-level reflector was employed to continuously generate low-frequency displacement to modulate the laser wave phase carrying the concentration of the Fe^2+^ indicator sample in the time domain. The high-reflecting plane mirror was employed to act as one side of the external cavity. Furthermore, the plane mirror could guarantee the homocentricity of the reflected wave, and the rays of the laser could be considered in the same direction, with the reflected rays persisting collinear to the optical axis of the laser propagation direction. Although the sample was diluted with water, there is only the absorption of Fe^2+^ since the absorption spectrum of Fe^2+^ lay in the green band, within which there was nearly no absorption of the water [42].

The Fe^2+^ indicator, a chemical complex Fe(C_12_H_8_N_2_)_3_^2+^ that can be obtained from the ferrous ion and the phenanthroline, served as a type of absorption sample in the experiment. The standard concentration of the solution was 0.02 mol/L and was diluted with pure water, and we determined the concentration with a photometer until the Fe^2+^ in the dilution reached the micromolar concentration.

The single longitudinal mode laser coupled with a fiber end of armor without isolators (SMI could not occur since an isolator might prevent the reflected light back into the laser cavity) acted as the light source of 4 mm diameter with 20 mW output at 532 nm, whose wavelength was located within the Fe^2+^ absorption spectrum. The loudspeaker was driven by a continuous sine function generated by a signal generator, whose amplitude and frequency were selected as 400 mVpp (in terms of μm magnitude) and 2 KHz, respectively. The absorption decay varied with the Fe^2+^ concentration of the indicator sample in the rectangular cuvette with a width of 1 cm, which was equal to the optical depth of the diluting sample. The laser was split into two beams by the optical splitter. One forward beam irradiated the mirror on the loudspeaker surface perpendicularly, and the backward reflected E_1_ and E_2_ waves after one round trip and two round trips turned back into the laser through the original path to generate the multiple self-mixing interference signal. Meanwhile, the alternating current (AC) photoelectric detector was used to monitor the other beam after magnification to obtain the multiple self-mixing interference signal. Because the laser wave phase was modulated versus time, the detected multiple self-mixing interference was also a time-domain signal, which could be obtained via an oscilloscope.

In order to analyze the experimental results, we derived the theoretical equations based on the three-mirror model [43,44,45] considering the absorption decay of the multiple reflected lights in the external cavity. To derive the lasing conditions in multiple self-mixing interference, including amplitude and phase conditions, the laser amplitude had to be considered with the forward and backward traveling waves after one round trip and two round trips in the external cavity [46]. The backward traveling waves injected lights back into the laser cavity, changing the laser amplitude by the gain coefficient *g*. If E_0_(t) accounts for the laser free amplitude without multiple self-mixing interference, the wave amplitude E_L_ in the laser cavity after one round trip can be expressed as

E_L_ = r_1_r_2_·exp[−*j*·4πL/λ) + *gL*]·E_0_, (1)

where the amplitude reflectivity r_1_ and r_2_ of the laser cavity consist of the input and output facets, *v* denotes the light frequency, φ_0_ is the initial phase, L is the laser cavity length, and λ is the light wavelength.

**Figure 2:**
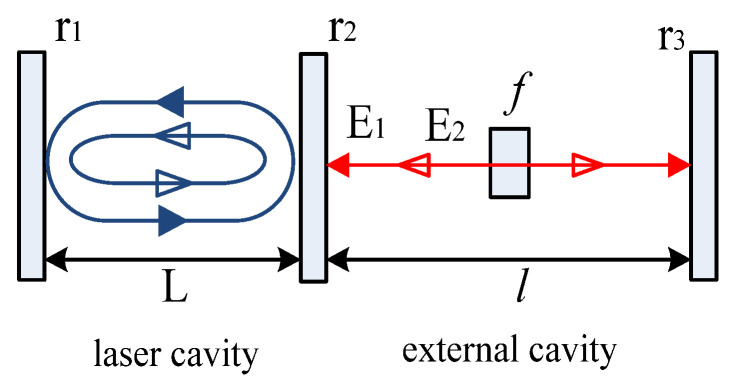
MSMI model with absorption dilution in an external cavity.

As shown in Figure 2, the reflected lights E_1_ and E_2_ were treated as a part of the laser cavity after multiple reflections. Therefore, the space between the output facet and the external reflector was treated as an external cavity according to the lasing condition. The absorption dilution in the external cavity would decrease the first and second reflected lights if the wave expression was multiplied by the amplitude parameter, indicating that the wave amplitude would be scaled up and down according to the value of this parameter. The wave amplitude E_1_ of the first reflected light after one round trip in the external cavity can be expressed as

E_1_ = r_1_(t_2_)^2^r_3_*f*^2^·exp[−*j*·4π(L + *l*)/λ) + *gL*]·E_0_, (2)

where *l* is the effective optical length of the external cavity, r_3_ is the amplitude reflectance of the external reflector, t_2_ is the amplitude transmittance of the output facet, and *f* is the amplitude transmittance of the absorption dilution in the external cavity.

The wave amplitude E_2_ of the second reflected light can be expressed by

E_2_ = r_1_r_2_(t_2_)^2^(r_3_)^2^*f*^4^·exp[−*j*·4π(L + 2*l*)/λ + *gL*]·E_0_, (3)

after two round trips in the external cavity. The varying laser amplitude E, considering multiple self-mixing interference, can therefore be expressed by

E = E_L_ + E_1_ + E_2_ = r_1_r_2_·exp[−*j*·4πL/λ) + *gL*]·E_0_ + r_1_(t_2_)^2^r_3_*f*^2^·exp[−*j*·4π(L + *l*)/λ) + *gL*]·E_0_ + r_1_r_2_(t_2_)^2^(r_3_)^2^*f*^4^·exp[−*j*·4π(L + 2*l*)/λ + *gL*]·E_0_. (4)

Once the laser output is steady, the laser with multiple feedbacks from the external cavity must satisfy the condition E = E_0_, yielding

exp[−*j*·4πL/λ) + *gL*]{r_1_r_2_ + r_1_(t_2_)^2^r_3_*f*^2^·exp[−*j*·4π*l*/λ) + r_1_r_2_(t_2_)^2^(r_3_)^2^*f*^4^·exp[−*j*·4π(2*l*)/λ]} = 1. (5)

We can define the phase shifting of one round trip in the external cavity as φ = 4π*l*/λ, with the amplitude coefficients of the first and second reflected lights as α = r_1_(t_2_)^2^r_3_*f*^2^ and β = r_1_r_2_(t_2_)^2^(r_3_)^2^*f*^4^ and obtain the amplitude condition

r_1_r_2_·exp[−*j*(4πL/λ) + *gL*][1 + α/r_1_r_2_·exp(−*j*φ_1_) + β/r_1_r_2_·exp(−*j*φ_2_)] = 1, (6)

where φ_1 =_ φ_0_ + φ and φ_2 =_ φ_0_ + 2φ denote the phases of E_1_ and E_2_, respectively. Because the external cavity will usually be tilted in the experiment to generate the MSMI waveform, φ_2_ will not be precisely equal to φ_1_. Therefore, the parameter φ_0_ was introduced to denote the phase resulting from the tilted external cavity, whose value would be assigned in the simulation to satisfy the experiment results.

The left term of Equation (6) above was complex while the right term was real, so the imaginary part of the left term had to be zero to satisfy the equation. To separate the real and imaginary parts of the left term, we assumed a complex parameter

z = Re(z) + *j*·Im(z) ={1 + α/r_1_r_2_·exp(−*j*φ_1_) + β/r_1_r_2_·exp(−*j*φ_2_)}, (7)

to account for the amplitude and optical path of the feedback wave with the real part Re(z) and imaginary part Im(z). The exponent terms in the equation corresponded to part of the wave phase in the exponential form, so we transferred the complex parameter into its triangular format. After the real and imaginary parts were separated, two expressions of *gL* and *θ* were obtained:

*gL* = −ln|r_1_r_2_(1 + α·exp(−*j*φ_1_) + β·exp(−*j*φ_2_))|/2. (8)

The real and imaginary parts were both defined to introduce the argument *θ* of z, to analyze the optical phase condition. The argument *θ* had to satisfy the following phase condition:

*θ* = arg(z) = arg[1 + α·exp(−*j*φ_1_) + β·exp(−*j*φ_2_)] = 2π−2πL/λ, (9)

to ensure exp[-*j*(4πL/λ + *θ*)] = 1. Thus, the complex parameter z was obtained precisely after the transformation of the exponent terms to triangle forms, and the equation was just derived on the condition of the imaginary part inside the exponent term and the real part outside the exponent term. In the logarithm of the equation, the expression of the gain coefficient *g* was obtained after derivation:

r_1_r_2_|z|·exp(*gL*)·exp{−*j*(4πL/λ + *θ*)} = 1. (10)

The argument *θ* of z depended on the real part Re(z) and imaginary part Im(z)

tan*θ* = Im(z)/Re(z) ≈ α*·*sin(−φ_1_) + β·sin(−φ_2_)/(r_1_r_2_), (11)

with the approximate conditions of r_1_r_2_ ≈ 1, α << 1, and β << 1, as the output facet was a high-level reflection mirror.

*θ* ≈ α·sin(−φ_1_) + β·sin(−φ_2_)/(r_1_r_2_). (12)

The argument *θ* was inserted into the expression

*gL*= −ln(r_1_r_2_ + r_1_r_2_α·exp(−*j*φ_1_) + r_1_r_2_β·exp(−*j*φ_2_))/2. (13)

thus,

*gL*= −ln(r_1_r_2_|z|)/2= −ln(r_1_r_2_) /2 −ln|z|/2, (14)

yielding

*gL*= −ln(r_1_r_2_)/2 − ln|z|/2= −ln(r_1_r_2_) /2 − [Re(z)]/2, (15)

with the approximate relation of {[Im(z)]^2^ + [Re(z)]^2^}^1/2^ ≈Re(z) if Re(z) >> Im(z) under the condition of α << 1 and β << 1, as mentioned above. Thus,

*gL*= −ln(r_1_r_2_)/2 − ln[Re(z)]/2= −ln(r_1_r_2_) /2 − [α·cosφ_1_/(r_1_r_2_) + β·cosφ_2_/(r_1_r_2_)]/2, (16)

with the approximate relation of

*g*= −ln[1 + α·cosφ_1_/(r_1_r_2_) + β·cosφ_2_/(r_1_r_2_)] = α·cosφ_1_/(r_1_r_2_) + β·cosφ_2_/(r_1_r_2_). (17)

Thus, we could obtain the cavity gain *g* with the multiple self-mixing interference

*g* = −ln(r_1_r_2_)/2*L* − [α·cosφ_1_/(r_1_r_2_) + β·cosφ_2_/(r_1_r_2_)]/2*L*. (18)

The free gain coefficient *g*_0_ could be obtained as *g*_0_ = −ln(r_1_r_2_)/2*L* without cosine feedback terms. Therefore, the influence of multiple self-mixing interference on the gain coefficient was estimated by Δ*g*, according to

Δ*g*= *g* − *g*_0_ = −[α·cosφ_1_/(r_1_r_2_) + β·cosφ_2_/(r_1_r_2_)]/2*L*. (19)

Finally, the varying lasing intensity *I* versus the free lasing intensity *I*_0_ with Δ*g* in the laser cavity could be expressed as

*I*/*I*_0_ = 1 − Δ*g*·2*L* = 1 + α·cosφ_1_/(r_1_r_2_) + β·cosφ_2_/(r_1_r_2_). (20)

The expression of *I* denoted the laser output intensity modulated by the feedback beams, which were related to the transmittance *f* of the Fe^2+^ indicator sample in the external cavity once the experimental setup was complete. Since the feedback beams passed through the Fe^2+^ indicator sample at different times during their round trips in the external cavity, the amplitude coefficients in the front of E_1_ and E_2_ had to depend on the absorption decay and the reflection decay in the external cavity. Therefore,

α = r_1_(t_2_)^2^r_3_
*f*^2^, (21)

and

β = r_1_r_2_(t_2_)^2^(r_3_)^2^*f*^4^, (22)

and the transmittance of the sample followed

*f* = 10^−^*^ε^*^bc^, (23)

according to Beer’s law, where *ε* is the molar absorption coefficient, and b and c are the optical depth and the concentration of the Fe^2+^ indicator sample, respectively.

In the expression, the phase *φ* of the beam that underwent one round trip could be expressed as

φ = 4π[*l* + Asin(*w*t)]/λ, (24)

where λ is the laser wavelength, and the initial phase of the wave was set to φ_0_ = −π/2 to make the simulated waveform close to the experimental waveform. Because the external cavity length varied slightly following the sine function Asin(*w*t) versus time *t* with amplitude A = 2 μm and frequency *w* = 2 kHz, the length of the external cavity *l* = 1.8 m for the phase φ was also modulated periodically by a sine-like signal in the time domain carrying the concentration c of the Fe^2+^ indicator sample.

## 3. Results

### 3.1. Simulated Results

To study the absorption decay in an external cavity, the waveforms of the varying lasing intensities *I* versus *I*_0_ at different Fe^2+^ concentrations c were simulated with the assigned parameters of r_1_ = 1.0, r_2_ = 0.97 [43], r_3_ = 0.99, b = 1 cm, and ε = 7955 L/(mol·cm). These parameters were assigned according to both the theoretical model and the experimental setup. The molar absorption coefficient of the diluent at the laser wavelength was estimated by measuring the diluent absorbance at different concentrations. For the laser cavity consisting of the input and output facets with reflectivity values of r_1_ and r_2_, we considered the input facet as a totally reflective mirror (r_1_ = 1) corresponding to the theoretical model without intensity loss in the inner cavity, while the output facet was a partially reflective mirror with a specific value of 0.97. The external reflector was a highly reflective mirror with a reflectivity of 0.99. If the absorption sample in the external cavity was the Fe^2+^ indicator whose concentration c ranged from 4.8 × 10^−6^ mol/L to 1.4 × 10^−5^ mol/L, the absorption decay depended on its molar absorption coefficient *ε* = 7955 L/(mol·cm) at the experimental laser wavelength and effective optical depth b = 1 cm, neglecting the intensity decay of the highly transparent container.

**Figure 3:**
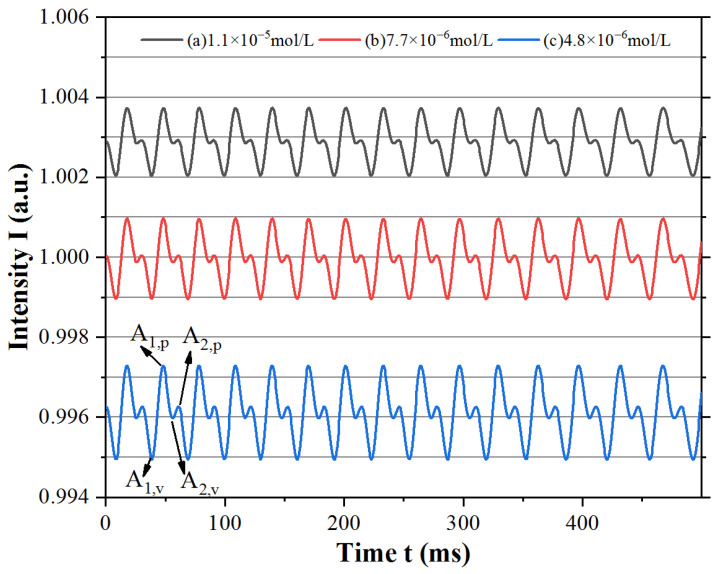
Simulated waveform at specific Fe^2+^ indicator concentrations: (**a**) 1.1 × 10^−5^ mol/L, (**b**) 7.7 × 10^−6^ mol/L, and (**c**) and 4.8 × 10^−6^ mol/L.

Because the phase φ was modulated periodically by the external reflector, the multiple self-mixing interference signals were in the time domain, but the amplitudes of the fringes were related to the concentration c, as shown in Figure 3. Meanwhile, Δg was related to the sum of feedback waves E_1_ and E_2_, which were decayed by the absorption in the external cavity. Thus, the feedback wave E_1_ caused some main fringes, while E_2_ caused some low parasitic fringes. Moreover, if the Fe^2+^ indicator was used as the absorption sample; it enabled the absorption decay for both E_1_ and E_2_ when the waves passed through the sample with different round trips. The peak values A_1,p_ and A_2,p_ and valley values A_1,v_ and A_2,v_ of the main and parasitic fringes were selected to obtain the amplitudes A_1_ and A_2_. The main and parasitic fringes were both decayed by the absorption of Fe^2+^ according to Beer’s law, and the parasitic fringe amplitude A_2,s_ = (A_2,p_ − A_2,v_)/2 decayed much more dramatically than the main fringe amplitude A_1,s_ = (A_1,p_ − A_1,v_)/2 with increasing concentration c of Fe^2+^ due to the difference in absorption decay between E_1_ and E_2_ in the external cavity.

### 3.2. Experimental Results

The multiple self-mixing interference signal was generated by the first and second reflected lights E_1_ and E_2_ from the external cavity. The reflected lights after one and two round trips entered back into the laser cavity and mixed with the wave field in the inner laser cavity. The waveform of the output laser consisted of the superposition of the two-fold signals, as the optical phase of E_2_ was approximately twice that of E_1_ after two round trips in the external cavity. As mentioned in Section 3.1, the self-mixing interference signal was affected by the Fe^2+^ indicator of the external cavity via the first and second reflected lights. Thus, the experimental signal at different absorption decays could be obtained by varying the Fe^2+^ concentration.

**Figure 4:**
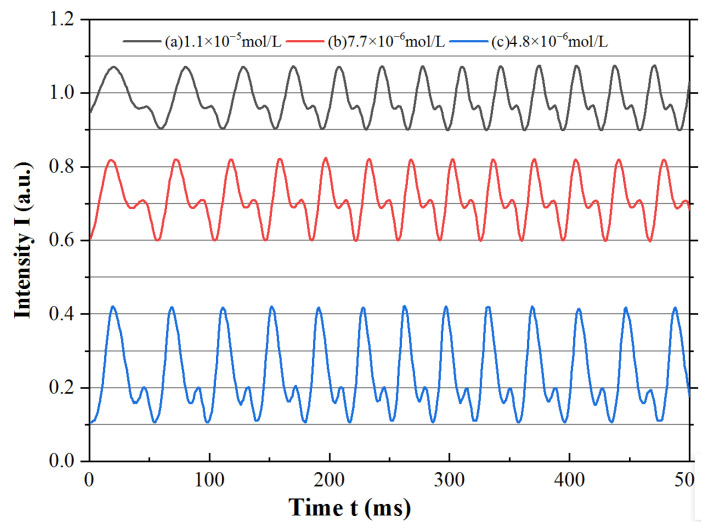
Experimental waveform at specific Fe^2+^ indicator concentrations: (**a**) 1.1×10^−5^ mol/L, (**b**) 7.7×10^−6^ mol/L, and (**c**) 4.8×10^−6^ mol /L.

We obtained experimental waveforms at different Fe^2+^ concentrations from 4.8 × 10^−6^ mol/L to 1.4 × 10^−5^ mol/L, as shown in Figure 4. When the concentration was quite low, such as approximately 10^−7^ mol/L, a change in A_2,e_/A_1,e_ could barely be obtained from the waveform. However, when the concentration reached a high value of more than 10^−5^ mol/L, the parasitic fringes became very imperceptible, and A_2,e_/A_1,e_ was nearly zero.

As shown in Figure 4, the specific self-mixing interference signal in the time domain showed that the waveform line of lasing intensity contained the main fringes and parasitic fringes, like the simulated waveforms. The numerical points of the experimental waveforms were obtained using the digital oscilloscope and selected the peak and valley values of the main and parasitic fringes to calculate the amplitudes A_1,e_ and A_2,e_ of the main and parasitic fringes. If the optical decay of the external cavity changed with the Fe^2+^ concentration, the amplitudes of the main fringes and parasitic fringes in the waveform line scaled up and down at different degrees as E_1_ and E_2_ underwent one and two round trips through the Fe^2+^ indicator, as the simulation results predicted.

We noticed that the waveform obtained in the experiment was not equal to the simulated waveform, as the experimental results were obtained after photoelectric conversion. The experimental results were electronic waveforms, while the simulated results were optical waveforms. There was a conversion coefficient of the photoelectric detector when the experimental results were transferred from optical signals to electronic signals, and the waveforms were scaled down overall. This meant that the amplitudes of the experimental main and parasitic fringes A_1,e_ and A_2,e_ were the products of the actual intensity and photoelectric conversion coefficient. Therefore, the amplitude ratio A_2,e_/A_1,e_ was employed to estimate the relationship between the waveforms and the concentration, as A_2,e_/A_1,e_ could eliminate the photoelectric conversion coefficient.

## 4. Discussion

**Figure 5:**
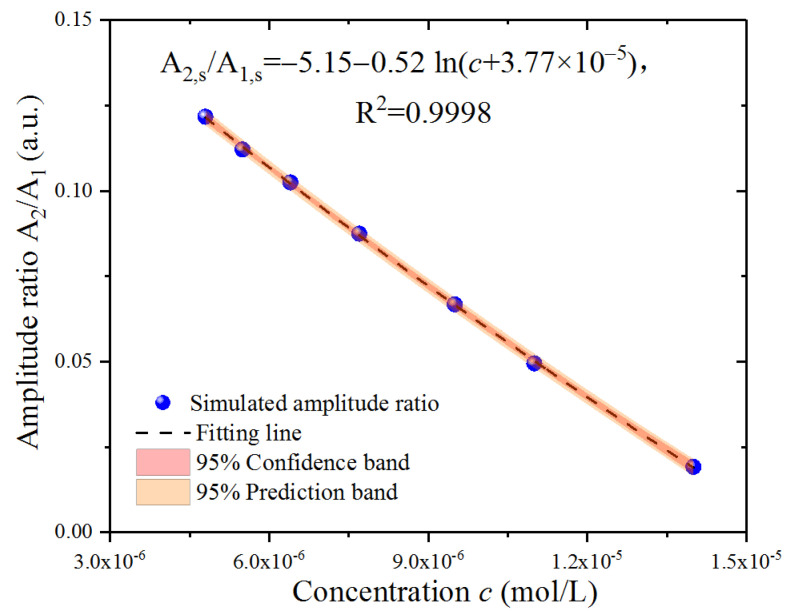
Numerical fitting of the simulation results around micromolar Fe^2+^ concentration.

To describe the simulation results, the amplitude ratio was defined as A_2,s_/A_1,s_, which gradually decreased with the Fe^2+^ concentration, as shown in Figure 5. Although the points in the figure show a linear trend, the distribution was not entirely linear. After multiple attempts of numerical fitting with the linear formula and the nonlinear logarithmic formula, we determined that a nonlinear formula could better fit the simulated A_2,s_/A_1,s_, based on the R^2^ of the fitting. The amplitude ratio A_2,s_/A_1,s_ was employed to fit with a concentration c, following the fitting logarithmic formula in the 95% confidence band and 95% prediction band, and the fitting result was A_2,s_/A_1,s_ = −5.15 − 0.52 ln (c + 3.77 × 10^−5^) with a goodness of fit R^2^ = 0.9998.

**Figure 6:**
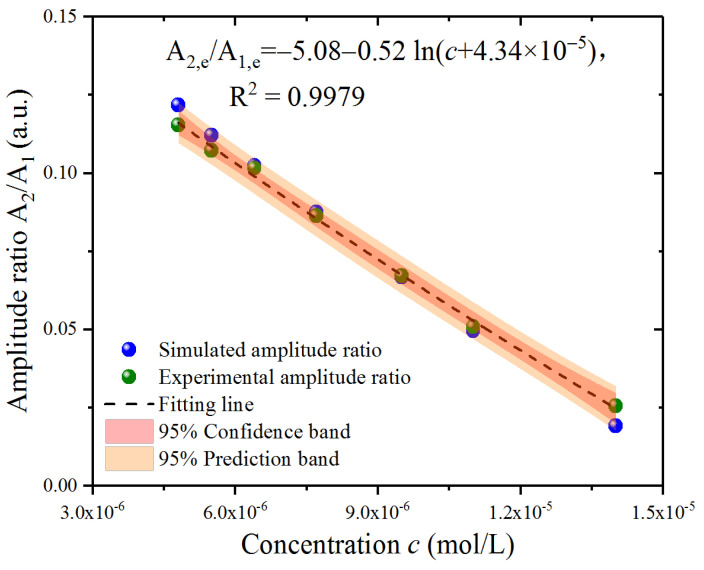
Numerical fitting of the experimental results around the micromolar Fe^2+^ concentration.

To estimate the relationship between the experimental results and the Fe^2+^ concentration, the points of the amplitude ratio A_2,e_/A_1,e_ around micromolar Fe^2+^ concentration are presented in Figure 6, with the simulated A_2,s_/A_1,s_ points at the same concentration also presented for comparison. The distribution was not entirely linear and was not as it appeared, similar to the fitting result in the simulation section. After the comparison of the fitting results using the linear and logarithmic formulas based on the fitting parameter R^2^, we determined that a nonlinear logarithmic formula might better fit the simulated A_2,s_/A_1,s_ since R^2^ = 0.9966 for linear fitting and R^2^ = 0.9979 for logarithmic fitting. The amplitude ratio A_2,s_/A_1,s_ was employed to fit with the concentration c following the fitting logarithmic formula in the 95% confidence band and 95% prediction band, and the fitting result was A_2,e_/A_1,e_ = −5.08 − 0.52 ln (c + 4.34 × 10^−5^) with a goodness of fit R^2^ = 0.9979. The goodness of fit indicated that the fitting logarithmic formula was suitable for both simulated and experimental results. In addition, the simulated results located in the prediction band calculated from the fitting results of the experimental results are shown in Figure 6.

**Figure 7:**
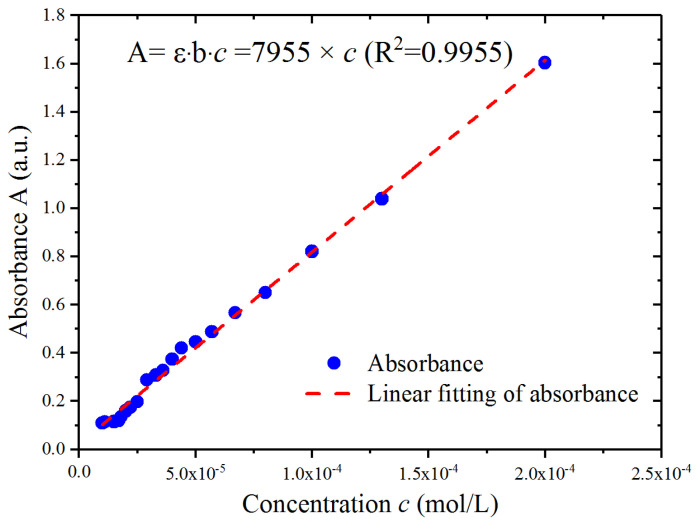
Absorbance of the Fe^2+^ indicator at different concentr1ations.

Typically, absorbance (A = lg(1/t) = *ε*·b·c), also known as the optical density, consists of the quantity of light absorbed by the Fe^2+^ indicator. We measured the output power to calculate the absorbance, as shown in Figure 7, before and after the laser passed through the Fe^2+^ indicator at different concentrations with the same laser in the experimental setup and with an optical power meter. Under the condition of b = 1 cm, the slope of the absorbance versus the Fe^2+^ concentration was estimated as the molar absorption coefficient *ε* = 7955 L/(mol·cm) at the wavelength of the laser. We noticed that the value of the optical power meter could barely be read when the concentration was less than 10^−4^ mol/L, as the meter was not sufficiently sensitive in this concentration regime. Furthermore, this made observations more difficult around 10^−6^ mol/L. We considered that the attributes of the reflected light carrying the absorption decay could be magnified in the laser cavity.

## 5. Conclusions

We proposed a novel method for measuring Fe^2+^ at a micromolar concentration based on multiple self-mixing interference. The absorption decay by the Fe^2+^ indicator in multiple self-mixing interference was observed, and we obtained its experimental waveforms around a micromolar concentration in an external cavity modulated by a periodic signal carrying the amplitude decay in the periodic form. The theoretical waveforms were simulated based on the lasing amplitude condition, considering multiple reflected lights. The experiment was conducted under the condition of Fe^2+^ indicator dilution at different concentrations in the external cavity. The waveform consisted of the main and parasitic fringes in the time domain and the amplitude decay of the fringes with different degrees, which was due to the absorption of Fe^2+^ indicator dilution. The experimental and simulated waveforms exhibited a similar trend when compared with the Fe^2+^ indicator concentration. Therefore, it was the absorption of the Fe^2+^ indicator that resulted in amplitude decay in multiple self-mixing interference.

This work assessed the combination of optical interference and the absorption spectrum, which could be useful for medical and environmental science or other optical spectrum cases. The absorption material corresponds to its spectral line, and nearly every laser will have a single mode or multiple modes, so a laser chosen according to the material’s absorption spectrum will be vital for successful experiments. Furthermore, the work employed a single longitudinal mode laser with a narrow spectrum band, which might only be useful for some specific types of electrolytes even if the absorption spectrum of the electrolyte lies around the laser wavelength. Therefore, we would have to find another laser with a different wavelength to detect another electrolyte if its absorption spectrum is not located in the same spectrum band. However, a kind of white laser with a wide spectrum band has been invented in recent years [47,48]; using this laser, more kinds of electrolytes will be detected in our future work.
